# Reactive Oxygen Species in Cardiovascular and Hematological Diseases: Mechanisms and Therapeutic Strategies

**DOI:** 10.7759/cureus.115330

**Published:** 2026-08-27

**Authors:** Anthony Alanis, Fernando Cisneros, Hector Trejo, Angela Hong, Kelsey Baker, Li Zuo

**Affiliations:** 1 Medicine, University of Texas Rio Grande Valley School of Medicine, Edinburg, USA; 2 Molecular Physiology, Princeton University, Princeton, USA; 3 Neuroscience, University of Texas Rio Grande Valley School of Medicine, Edinburg, USA; 4 Physical Therapy and Medical Education, University of Texas Rio Grande Valley School of Medicine, College of Health Professions, Edinburg, USA

**Keywords:** antioxidants, cardiovascular disease, crispr, genetic engineering, hematological disorder, nanotechnology, oxidative stress

## Abstract

Reactive oxygen species (ROS) play a vital role in normal cellular functions, including cellular signaling and metabolism of toxins. Excessive ROS contributes to the pathogenesis of cardiovascular and hematologic conditions by altering genetic expression, signal transduction, lipid peroxidation, and vascular remodeling. This article investigates the role of ROS in diseases such as cardiovascular disorders and hematological conditions, focusing on molecular mechanisms and emerging treatments for preventative and acute management. We analyzed the role of ROS in mitochondrial dysfunction, redox signaling pathways, and their contribution to inflammation and tissue damage across cardiovascular and blood disorders. We also discuss novel therapies targeting oxidative stress, such as antioxidants, mitochondrial agents, nanomedicine, and clustered regularly interspaced palindromic repeats. Particularly, in chronic lymphocytic leukemia, ROS-driven mitochondrial bioenergetics and redox pathways are exploited to trigger apoptosis, presenting opportunities for therapeutic intervention. Our paper indicated that ROS exhibits a paradoxical nature - both beneficial and harmful - offering a unique opportunity for therapeutic exploration. Redox therapies, targeted antioxidants, and gene-editing technologies can improve patient outcomes in both cardiovascular and hematological diseases.

## Introduction and background

Reactive oxygen species (ROS) are a collective term for a group of very reactive oxygen-derived molecules. They are produced from several cellular sources, including complexes I and III of the mitochondrial electron transport chain, nicotinamide adenine dinucleotide phosphate (NADPH) oxidases (NOX) in cell membranes, and via the β-oxidation of fatty acids in peroxisomes. Under normal physiological conditions, ROS play essential roles as redox signaling molecules, participating in kinase signaling pathways and programmed cell death. The generation of ROS is balanced by a set of antioxidant defense systems, including the enzymes superoxide dismutase (SOD) and glutathione peroxidases (GPx), and non-enzymatic antioxidants like glutathione (GSH), vitamin C, and vitamin E. When the production of ROS exceeds the capacity of the antioxidant defense, the imbalance causes a state defined as oxidative stress (OS).

OS can result in indiscriminate, wide-spectrum damage to macromolecules like proteins, enzymes, lipids, membranes, and nucleic acids. Such damage is implicated in the pathogenesis of a myriad of diseases and can ultimately result in cell death by apoptosis [1]. ROS have been found to influence the pathogenesis of various cardiovascular and hematological diseases. Mechanisms by which ROS influence the pathogenesis of cardiovascular disease often involve direct oxidation reactions, biochemical changes, and physiological alterations. Direct oxidation reactions include the peroxidation of lipids on endothelial cell membranes, causing atherosclerosis. Biochemical changes include the impairment of nitric oxide (NO) bioavailability, leading to hypertension [2]. Physiological alterations include the activation of cardiac sodium-hydrogen exchanger 1 (NHE1), resulting in cardiac hypertrophy [3]. Other vital mechanisms include the surge of ROS during reperfusion that injures blood vessels and organs [4], the release of vasoconstricting agents from vascular endothelia during oxidative stress (OS) [5], and the exacerbation of clot formation during OS via activation of platelet-derived growth factor, angiotensin II, and prostaglandin F2 [6].

Hematological diseases, such as anemia, leukemia, and myelodysplastic syndrome (MDS), are often triggered by direct or indirect oxidation of DNA. As such, single and double-strand DNA breaks and various oxidized nucleotides are highly likely [7,8]. In diseases such as hemophilia, OS may arise as a downstream complication. Through the activation of p38, ROS impairs platelet function and triggers them to shed key adhesion receptors [9]. Additionally, ROS appear to alter RBC membrane fragility, increasing their recognition by reticuloendothelial macrophages and improving their clearance [10]. OS may induce apoptosis in leukemia cells through both extrinsic and intrinsic pathways [11]. Lastly, ROS can trigger clonal expansion of hematopoietic stem cells, leading to MDS [8].

Therapeutic strategies targeting OS in cardiovascular diseases involve agents that modulate ROS levels by either increasing or decreasing ROS. These agents include mitochondrial-targeted compounds, antioxidant vitamins, antioxidant enzymes, pharmacological agents, nanoparticles, and chelating agents. Many of these have been studied for their use in treating multiple ROS-involved cardiovascular diseases; however, some agents have specific uses in certain diseases. Among the therapies involved in treating OS in hematological diseases, many of them include the addition of targeted pro-oxidants, enzyme and signal transduction modulators, immunomodulatory and anti-inflammatory agents, and cellular therapy/transplantation. In addition to redox therapies, nanotechnology has proven to be an effective tool for delivering therapeutics to impacted areas, such as to mitigate the infarct volume from ischemic stroke [12]. Other novel techniques being investigated to combat OS in sickle cell disease (SCD) include genetic engineering. Although all the previously mentioned therapies are different in their mechanisms of treating OS, which will be elaborated throughout the paper, they all can either increase, decrease, or prevent OS from developing.

Over recent years, the investigation into redox therapies has increased in the management of the diseases above. However, many of these therapies have yet to be studied in a clinical setting. Thus, it is essential to provide a timely review of these diseases from a redox perspective.

## Review

ROS in cardiovascular disease

As previously stated, ROS have been found to impact various cardiovascular conditions. An imbalance of ROS is a common etiology for many of these conditions, driven by direct oxidation reactions, biochemical changes, and physiological alterations. One source of ROS is nicotinamide adenine dinucleotide phosphate oxidase (NOX), which can provoke inflammatory endothelial damage, lipid peroxidation, and vascular remodeling in the heart. Vascular remodeling, in this case, involves the processes dysregulating normal cellular proliferation, apoptosis, senescence, cell differentiation, and cell cycle regulation [13]. Many other disease-specific mechanisms are implicated in the pathogenesis of cardiovascular diseases. Consequently, redox therapies have been developed to combat inflammation and improve clinical outcomes.

ROS in peripheral vascular disease

**Figure 1:**
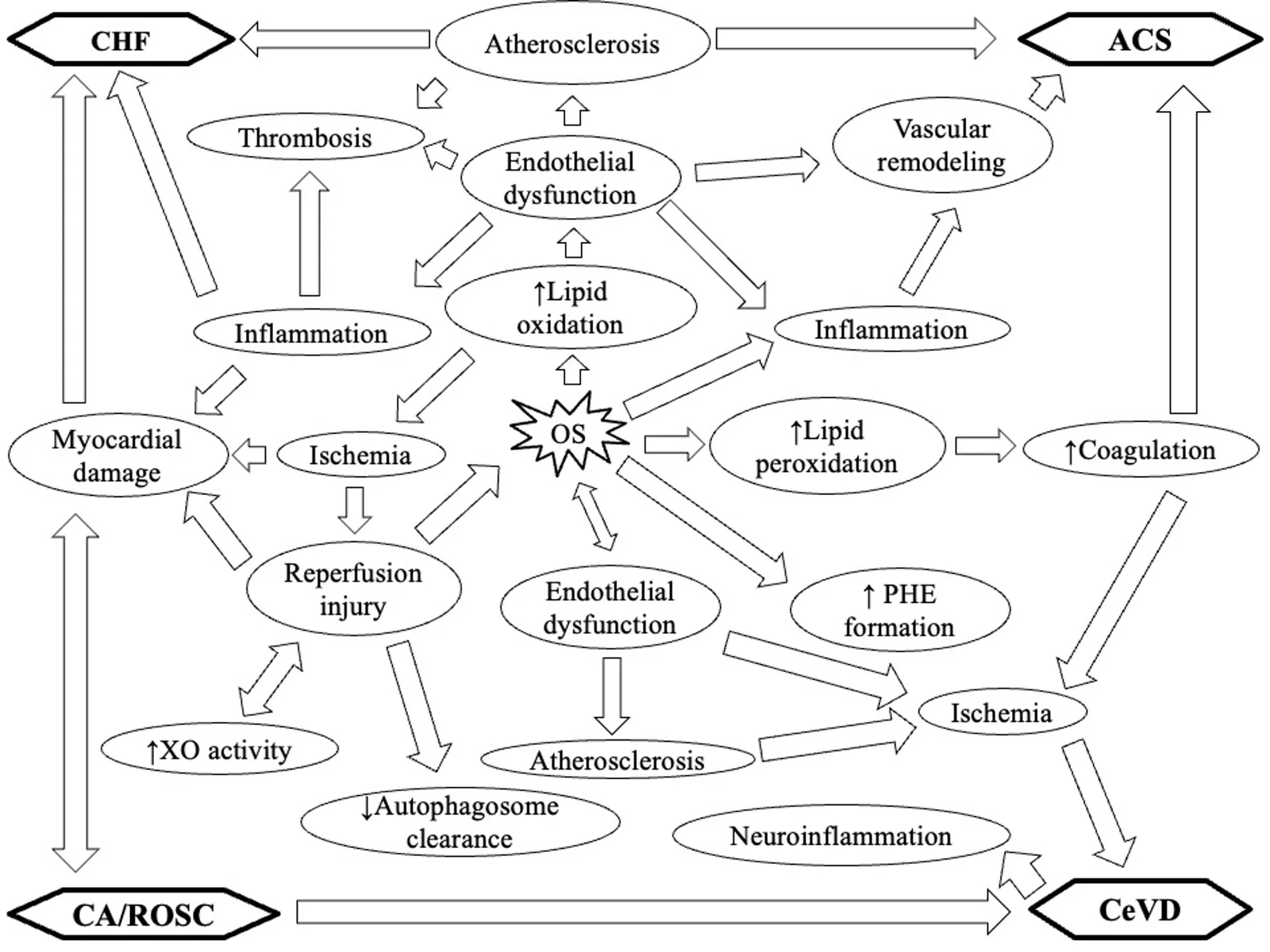
Organizational chart of ROS in cardiovascular diseases focusing on pathogenesis Figure created by the authors using PowerPoint (Microsoft, Redmond, Washington). ROS - reactive oxygen species; ACS - acute coronary syndrome; CA/ROSC - cardiac arrest/ return of spontaneous circulation; CeVD - cerebrovascular disease; CHF - congestive heart failure; PHE - perihematomal edema; OS - oxidative stress; XO - xanthine oxidase

Peripheral vascular disease (PVD) refers to a condition in which there is a buildup of fatty deposits (atherosclerosis) in arteries that perfuse the extremities. Atherosclerosis in the extremities can lead to symptoms of claudication, such as pain, cramps, or paresthesia distal to the site of occlusion. Transient ischemia and subsequent reperfusion during exercise can increase OS and cause endothelial dysfunction in the lower extremities [14]. When the body is experiencing OS, excess ROS can oxidize lipids, proteins, and DNA throughout the cardiovascular system. This causes damage to the endothelium, the underlying substrate for vascular dysfunction, which is a key mechanism in the development of atherosclerosis and PVD. Oxidized low-density lipoproteins (LDLs), in particular, activate lectin-like oxidized LDL receptors, which has detrimental effects on the endothelium, including impaired NO bioavailability and promotion of a prothrombotic surface [15]. Furthermore, the nonenzymatic glycosylation of proteins during chronic hyperglycemia, commonly seen in diabetics, can trigger the formation of advanced glycation end-products (AGEs). Excess AGEs cause OS in vascular cells primarily via the receptor for AGEs (RAGE), which activates NOX to form ROS and alter gene expression. AGEs can also inactivate NO, causing impaired vasodilation and vascular dysfunction. Additionally, AGE/RAGE signaling contributes to mitochondrial ROS formation, exacerbating OS (Figure 1) [16].

Therapies for ROS in Peripheral Vascular Disease

Patients with PVD commonly experience ischemic episodes requiring reperfusion via percutaneous transluminal angioplasty (PTA). After reperfusion, a surge of ROS can occur due to mitochondrial dysfunction and the rapid influx of oxygen. Activated leukocytes also produce ROS as a defense mechanism, further contributing to ischemia-reperfusion injury (IRI). A double-blinded, single-center randomized controlled trial was conducted on 94 hospitalized patients with symptomatic lower-extremity arterial disease (LEAD) scheduled for lower-limb digital subtraction angiography (DSA). The study showed that ischemic preconditioning (IPC), involving short, repetitive episodes of ischemia induced with a blood pressure cuff, can be used to temper the amount of oxidative damage experienced from IRI in patients with LEAD undergoing DSA and DSA-PTA. The isoprostane-creatinine ratio (a measure of lipid peroxidation) was significantly lower in the IPC group compared to the non-IPC group, suggesting that the IPC group experienced less OS and vascular damage. Additionally, an anti-inflammatory plasma protein called adiponectin (which is released from adipose tissue) was found to be significantly elevated in the non-IPC group, suggesting higher levels of inflammation. These findings support the idea that IPC could perhaps be a non-invasive therapeutic technique for combating the damaging effects of reperfusion using PTA. However, other OS markers that were measured, such as myeloperoxidase (MPO) and oxidized LDL, did not show significant differences between groups 24 hours after reperfusion. Furthermore, IPC failed to show clinically significant benefits in improving renal biomarkers, such as creatinine, estimated glomerular filtration rate (eGFR), and cystatin C. It also failed to improve cardiac markers, including hs-troponin-T and NT-proBNP, between IPC and sham groups. Therefore, more investigation is needed to uncover whether IPC is beneficial for patients with LEAD undergoing DSA or DSA-PTA [4].

Multiple classes of systemic redox therapies are being explored for their beneficial effects on the vascular endothelium. Antioxidant enzymes, such as superoxide dismutase (SOD), hemeoxygenase-1, and GPx, have been shown to mitigate OS and improve peripheral vascular function. Modern antidiabetic drugs, such as sodium/glucose cotransporter 2 (SGLT2) inhibitors (e.g., empagliflozin), dipeptidyl peptidase 4 (DPP-4) inhibitors (e.g., linagliptin), and GLP-1 analogs (e.g., liraglutide, semaglutide), are associated with reduced OS and improved vascular function in rat models. Furthermore, while GLP-1 agonists and DPP-4 inhibitors exhibit some cardioprotective effects and strong anti-inflammatory properties, SGLT-2 inhibitors are particularly highlighted for their benefits, as further demonstrated in animal experimental models of diabetes and sepsis. GLP-1 agonists are able to reduce OS by elevating cyclic AMP levels, activating protein kinase A, and inhibiting monocytic oxidative burst. They also improve endothelial function through the reduction of nitro-OS, such as 3-nitotyrosine formation [17].

This growing knowledge highlights the potential for non-invasive interventions to mitigate disease progression (e.g., IPC). Accordingly, redox therapy holds promise for becoming a highly effective treatment strategy for cardiovascular disease. However, there are insufficient clinical trials with human subjects to confirm the clinical benefits of redox therapies in PVD. More investigation is needed to understand the role of redox therapy in PVD.

ROS in congestive heart failure

Congestive heart failure (CHF) is a syndrome characterized by cardiac dysfunction, typically from myocardial damage, leading to left ventricular changes, fluid retention, and circulatory abnormalities [18]. CHF can be simplified into two types: systolic heart failure, when there is insufficient blood being ejected from the cardiac ventricles during systole, and diastolic heart failure, when there is insufficient blood filling the cardiac ventricles during diastole [19]. OS contributes to the pathogenesis of CHF by triggering inflammation within the heart, causing myocardial damage and adverse cardiac remodeling [20-22]. In a study on myocardial infarction (MI)-induced rats, cardiac remodeling was attributed to OS resulting from increased Ca²⁺ influx and protein alterations [23]. OS can also cause endothelial damage and increase vascular stiffness, which prevents the ventricles from relaxing properly, causing diastolic heart failure (Figure 1).

ROS have links to the development of diseases leading to CHF, such as hypertension and ischemic heart disease, through modulation of vasoactive substances, like NO [24]. Additionally, ROS are infamous for their ability to oxidize lipids, including those on the membranes of cardiomyocytes, which compromises their function and contractile force [25]. ROS can modify LDLs in the coronary arteries, which are taken up by macrophages, forming foam cells. This process contributes to endothelial dysfunction, inflammation, atherosclerosis, and thrombosis, worsening CHF outcomes [26]. Other than lipid oxidation, ROS can trigger vascular smooth muscle cells (VSMCs) to constrict, resulting in altered blood flow and contributing to atherosclerotic plaque formation [27]. The disruption of an atherosclerotic plaque exposes thrombogenic components, also triggering platelet aggregation and coagulation, which together form a stable thrombus (Figure 1) [28].

Physiologically, cardiac sodium-hydrogen exchanger 1 (NHE1) on cardiomyocytes is activated by ROS to initiate the growth of cardiac muscle tissue (cardiac hypertrophy). ROS also promote the activation of Ca2+/calmodulin-dependent protein kinase II (CaMKII), which causes the death of cardiomyocytes and is associated with reduced ejection fraction and progression of CHF. Activation of CaMKII also results in elevated sodium levels within cardiomyocytes, which may contribute to impaired cardiac muscle relaxation and the onset of arrhythmias. In addition to NHE1, ROS can suppress sarcolemma sodium-potassium ATPase, further contributing to cardiac hypertrophy [3].

Therapies for ROS in CHF

Multiple compounds are being studied for their ability to protect the heart from ROS generated during IRI, a major contributor to the development of CHF. Mitochondrial agents, such as the mitochondrial-targeted peptide elamipretide (SS-31), interact with cardiolipin, a lipid on the inner mitochondrial membrane critical for the assembly of respiratory complexes in the electron transport chain (ETC). In Dawley rat models, the binding of SS-31 to cardiolipin prevents its oxidation and protects against mitochondrial OS. Another mitochondrial agent, mitoquinone (MitoQ), improves left ventricular dysfunction, mitochondrial structure, and reduces cardiac fibrosis in heart failure models by regulating mitofusin-2 [29] and inhibiting key profibrogenic pathways [30]. MitoQ also alleviates chronic inflammation in metabolic syndrome by modulating oxidized mitochondrial DNA and the TLR9-NF-κB pathway [31].

**Figure 2:**
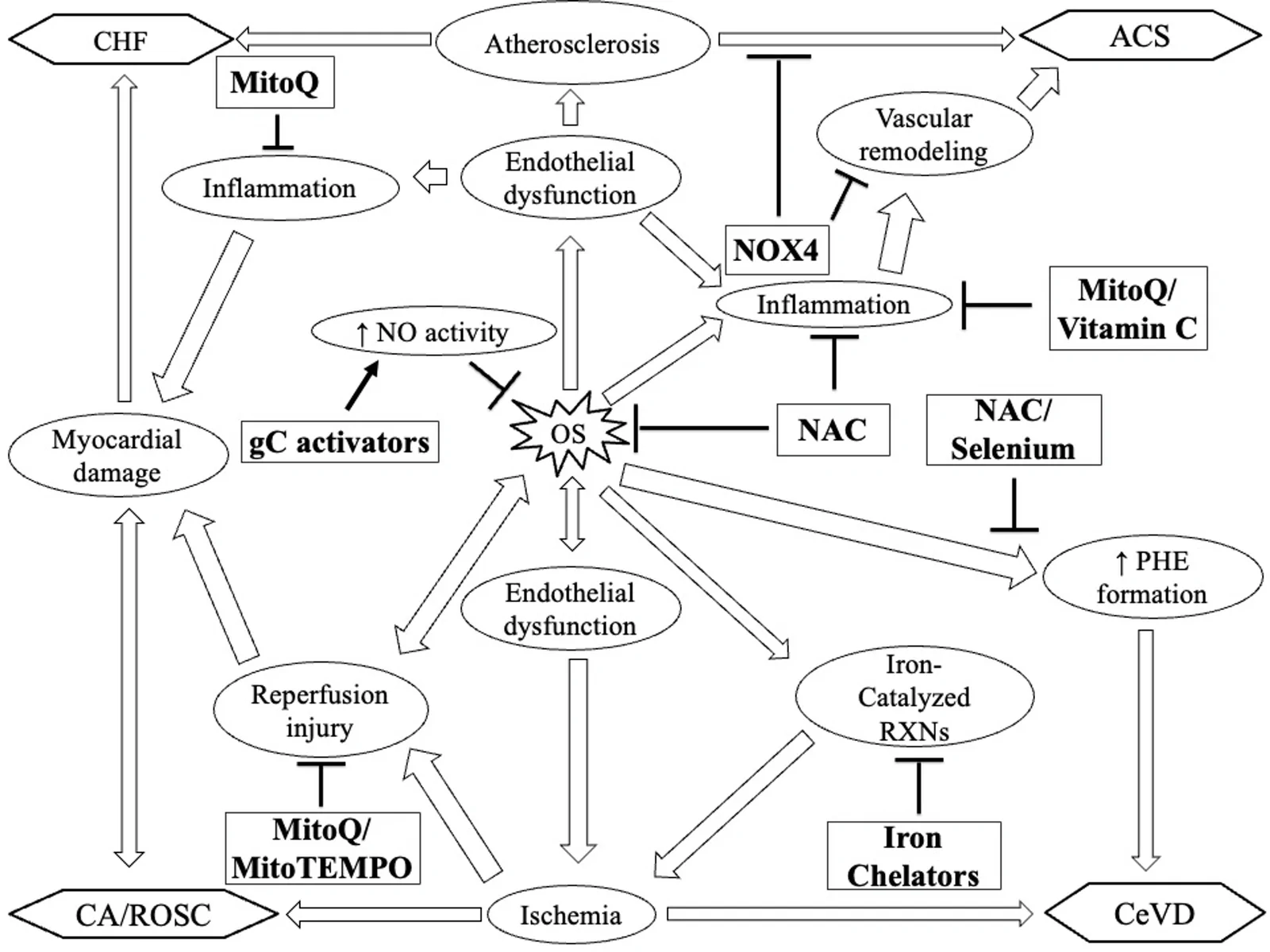
Organizational chart of ROS in cardiovascular diseases focusing on therapeutics Figure created by the authors using PowerPoint (Microsoft, Redmond, Washington). ROS - reactive oxygen species; ACS - acute coronary syndrome; CA/ROSC - cardiac arrest/ return of spontaneous circulation; CeVD - cerebrovascular disease; CHF - congestive heart failure; gC - guanylyl cyclase; ICH - intracerebral hemorrhage; MitoQ - mitoquinone; MitoTEMPO - 2,2,6,6-tetramethyl-4-((2-(triphenylphosphonio) acetyl) amino)-1-piperidinyloxy, monochloride, monohydrate; NAC - N-acetylcysteine; NO - nitric oxide; NOX - nicotinamide adenine dinucleotide phosphate oxidase; OS - oxidative stress; PHE - perihematomal edema; RXNs - reactions

Guanylyl cyclase activators and stimulators, BAY 58-2667 and BAY 60-2770, modulate OS by enhancing NO signaling, thereby reducing ROS production as demonstrated on human α1 and β1 subunits (Figure 2) [32]. In a separate study, a combination therapy of hydralazine and nitrates has been shown to increase survival in patients with advanced heart failure. This effect is achieved by balancing NO production from nitrates and reducing superoxide formation. Elevated levels of NO also help reduce afterload on the heart, lowering strain on the heart during systole [21]. Moreover, combination therapies, such as hydralazine and isosorbide dinitrate, reduce OS and improve heart failure outcomes through effects on enhancing NO bioavailability, reducing preload and afterload through vasodilatory actions, and mitigating OS [33].

ROS in acute coronary syndrome

Ischemic heart disease is a condition where reduced blood flow to the heart leads to an imbalance between the heart's oxygen supply and its oxygen needs [34]. The results of OS in the development of vasoconstriction, platelet aggregation, and inflammation have similar mechanisms as discussed in the development of CHF [35]. Ischemia often occurs as a result of coronary atherosclerosis, which develops due to lipoprotein deposition in the arterial wall, leading to obstruction [36]. When an obstruction to blood flow occurs in the heart, ROS are generated by a variety of NOX isoforms, which are a subset of enzymes in the NOX family, provoking inflammatory endothelial damage, lipid peroxidation, and vascular remodeling. Oxidized LDLs become internalized by arterial wall macrophages, forming foam cells and atherosclerotic plaques. ROS-mediated damage to endothelial cells also invites immune cells to attack by increasing the expression of surface adhesion molecules [13]. This is accompanied by proinflammatory cytokines such as IL-1 and interferon, exacerbating clot formation and inflammatory cell recruitment [6]. During acute coronary syndrome (ACS), ROS are generated by NOX isoforms, provoking inflammatory endothelial damage, lipid peroxidation, and vascular remodeling in the heart [13]. 

Coagulation-independent mechanisms of vascular injury have been described via vascular remodeling of human VSMCs. Protease-activated receptors (PAR)-2 have been demonstrated to participate in vascular remodeling during vascular injury. In atherosclerotic human vascular smooth muscle cells, FXa markedly elevates NOX-1 expression, producing nearly a fourfold rise in NOX-1 mRNA after 1 hour and a threefold increase in total protein after 24 hours. Furthermore, NOX-1 knockdown abolishes PAR-2 expression, suggesting that NOX-1-containing oxidases regulate PAR-2 expression [37]. In a separate study, Johny et al. observed the formation of platelet-monocyte aggregates (PMAs) in diabetic patients and how it contributes to the risk of coronary artery disease (CAD). One hundred twenty-two patients from a hospital in Guwahati, Assam, were recruited for this study and separated into four groups. The groups consisted of patients without diabetes, patients with a diagnosis of only type 2 diabetes (HbA1c ≥6.5%), patients with a diagnosis of only CAD, and patients with a diagnosis of both CAD and type 2 diabetes. They found that high glucose levels promote the formation of PMAs, which are involved in the development of CAD (Figure 1) [38].

Alternatively, a closely related enzyme, NOX4, appears to protect the endothelial lining of cardiac blood vessels from ROS-mediated plaque formation. In a study conducted on mice with hypertension, it was concluded that NOX4 promoted eNOS activation and NO release from endothelial cells [39]. Furthermore, it was observed that levels of NOX4 mRNA are reduced in both human and mouse arterial plaques [40]. Additionally, studies on NOX4 knockout mice have revealed a notable increase in plaque area accompanied by a corresponding decrease in H2O2 levels, reinforcing the idea that NOX4 has an atheroprotective role [40,41] (Figure 2). Despite the atheroprotective properties of H2O2, at high levels, the formation of ROS can uncouple eNOS by oxidizing tetrahydropiopterin, an essential cofactor for eNOS. This causes eNOS to produce superoxide instead of NO (a vasodilator), leading to vasoconstriction and vascular inflammation, two key mechanisms in the pathogenesis of atherosclerotic plaque formation and ACS [42]. In addition to the decreased atherosclerotic effects, NOX4 also reduces vascular remodeling by inhibiting VSMC proliferation and maintaining eNOS expression, further supporting its beneficial effects in ACS development (Figure 2) [13]. Peroxidized lipids generate 4-hydroxy-2-nonenal, which promotes the release of tissue factor microvesicles from perivascular cells as reported in a study on mice [43]. Tissue factor, in turn, enhances the coagulation cascade by facilitating thrombin formation [44]. A study by Loeffen et al. found significantly higher levels of thrombin generation, factor XIa, and D-dimer in ACS patients compared to non-ACS patients, indicating their potential as predictors of recurrent cardiovascular events (Figure 1) [45].

Therapies for ROS in ACS

Redox therapies designed to mitigate OS during myocardial ischemia reperfusion injury (MIRI) have been developed with many different approaches. ROS-responsive biomaterials, including NPs, injectable hydrogels, and biomimetic biomaterials, offer targeted modulation of OS. In a study on H9C2 cardiac cells, mitochondria-targeted nanomicelles and ROS-responsive covalent organic frameworks support tissue repair and vascular regeneration and function as pro-survival and pro-angiogenic therapies [46]. Vitamin C and NAC have proven beneficial in preclinical studies [47]. Vitamin C's anti-inflammatory effects were highlighted in a systematic review of randomized controlled trials, which showed that intravenous (IV) vitamin C administration before percutaneous coronary intervention (PCI) reduced cardiac injury biomarkers, such as troponin, CK-MB [48]. NAC not only reduces inflammation by inhibiting ROS but also decreases inflammatory cytokine activation of nuclear factor kappa-light-chain enhancer of activated B cells (NF-κB), lowering levels of IL-6, IL-10, and TNF-α as demonstrated in sepsis-induced rats [49].

As mentioned previously, MitoQ specifically targets mitochondrial ROS, effectively reducing myocardial damage in preclinical models by suppressing inflammation through decreased cytokine levels and inhibiting cytokine maturation (Figure 2) [50,51]. MitoQ also alleviates the severity of CHF by preserving mitochondrial network integrity in a pressure-overload heart failure model, by regulating redox-related noncoding RNAs, and maintaining the expression of mitochondrial fusion proteins such as mitofusin-2 (MFN2). Particularly, MFN2 plays a critical role in CHF because its overexpression enhances the subunits of respiratory chain complexes and oxidative phosphorylation, while its repression impairs glucose oxidation. MitoQ improves MFN2 expression and mitochondria-SR coupling, alleviating calcium dysregulation in heart failure [29].

ROS in cardiac arrest/return of spontaneous circulation

Cardiac arrest (CA) signifies the abrupt halt of the heart's ability to eject blood, resulting in the cessation of blood flow to essential organs. Return of spontaneous circulation (ROSC) marks the return of effective heartbeats without external assistance after CA, typically following interventions such as cardiopulmonary resuscitation and defibrillation [52]. Emerging evidence suggests that ROS accumulate swiftly during the onset of ischemia in striated muscle. During this phase, XO appears to be the primary source of ROS. During reperfusion, XO catalyzes the formation of xanthine and superoxide [53]. Reperfusion causes massive ROS production, which induces cell death in ischemic tissues through DNA damage, protein carbonylation, and lipid peroxidation [54]. Neutrophil recruitment augments ischemic damage by producing ROS via NOX enzymes, causing lipid peroxidation [55]. Moreover, ROS can instigate a proinflammatory response, which can exacerbate pre-existing cardiac muscle damage and worsen patient outcomes. ROS generated during reperfusion induce a robust proinflammatory reaction, exacerbating muscle damage. There can also be dysfunctional autophagosome-lysosome fusion during reperfusion, resulting in apoptosis of cardiomyocytes. During MIRI, lysosomal dysfunction or autophagosome maturation defect blocks their fusion, resulting in arrested autophagic flux, protein accumulation, disruption of cellular homeostasis, and impairment of cell function [56]. Studies in rat cardiomyocytes also suggest the activation of the lysosomal channel TRPML1 by elevated ROS in MIRI. This activation leads to the release of lysosomal zinc into the cytosol, which is most likely to disrupt autophagosome-lysosome fusion and autophagy (Figure 1) [57].

Therapies for ROS in CA/ROSC

Redox therapies, including vitamin C, improve outcomes after cardiopulmonary resuscitation. In a study on Sprague-Dawley rats treated with vitamin C after cardiopulmonary resuscitation, there was an improvement in ejection fraction, cardiac output, and myocardial performance index versus a control. Additionally, there was a decrease in oxidative damage and inflammatory markers (e.g., IL-6, TNF-) in cardiomyocytes after cardiopulmonary resuscitation. It should be noted that this study was conducted on mice without any underlying heart diseases [58].

Deferoxamine, an iron chelator, has also been studied for its capacity to reduce ROS formation by limiting iron-catalyzed reactions, aiding in functional recovery in animal models with myocardial ischemia followed by ROSC. Cellular death during an ischemic episode releases ferric iron (Fe3+) that can create ROS through Fenton reactions, which produce toxic quantities of superoxide and hydroxyl radicals as byproducts while reducing Fe3+ to ferrous iron. Additionally, in a study using isolated, perfused rabbit hearts, deferoxamine, given either during ischemia or at the start of reperfusion, significantly improved heart function and energy metabolism compared to controls. Hearts treated with deferoxamine during ischemia also showed better recovery of developed pressure and prevented a spike in free radical production during reperfusion [59]. In a separate study using a mouse model of MIRI, it was reported that treatment with deferasirox can reduce heart damage, lower iron overload in the endoplasmic reticulum, and prevent ferroptosis in both cultured heart cells and I/R-injured heart tissue [60].

2,2,6,6-tetramethyl-4-((2-(triphenylphosphonio) acetyl) amino)-1-piperidinyloxy, monochloride, monohydrate (MitoTEMPO), another mitochondrial agent, has also shown significant promise for improving patient outcomes from ischemic reperfusion injuries. MitoTEMPO evidently protected the heart from oxidative damage and cardiac dysfunction in middle cerebral artery occlusion stroke models. The antioxidant effect that MitoTEMPO has on the cardiovascular system stems from its ability to target a G-protein coupled receptor signaling process in mitochondria. This inhibition can help optimize mitochondrial homeostasis, excessive mitochondrial fission, and cell death during an ischemic episode (Figure 2) [61]. These findings suggest that addressing OS and mitochondrial dysfunction could enhance recovery. However, more evidence is needed to validate the therapeutic benefits of mitochondrial-targeted agents. Therefore, redox therapies addressing CA and ROSC include nonspecific antioxidants (e.g., vitamin C) and targeted antioxidants (e.g., MitoQ, MitoTEMPO). These advancements illuminate a future where redox therapies are at the forefront of cardiac care; however, human trials are still very limited.

ROS in cerebrovascular disease

**Figure 3:**
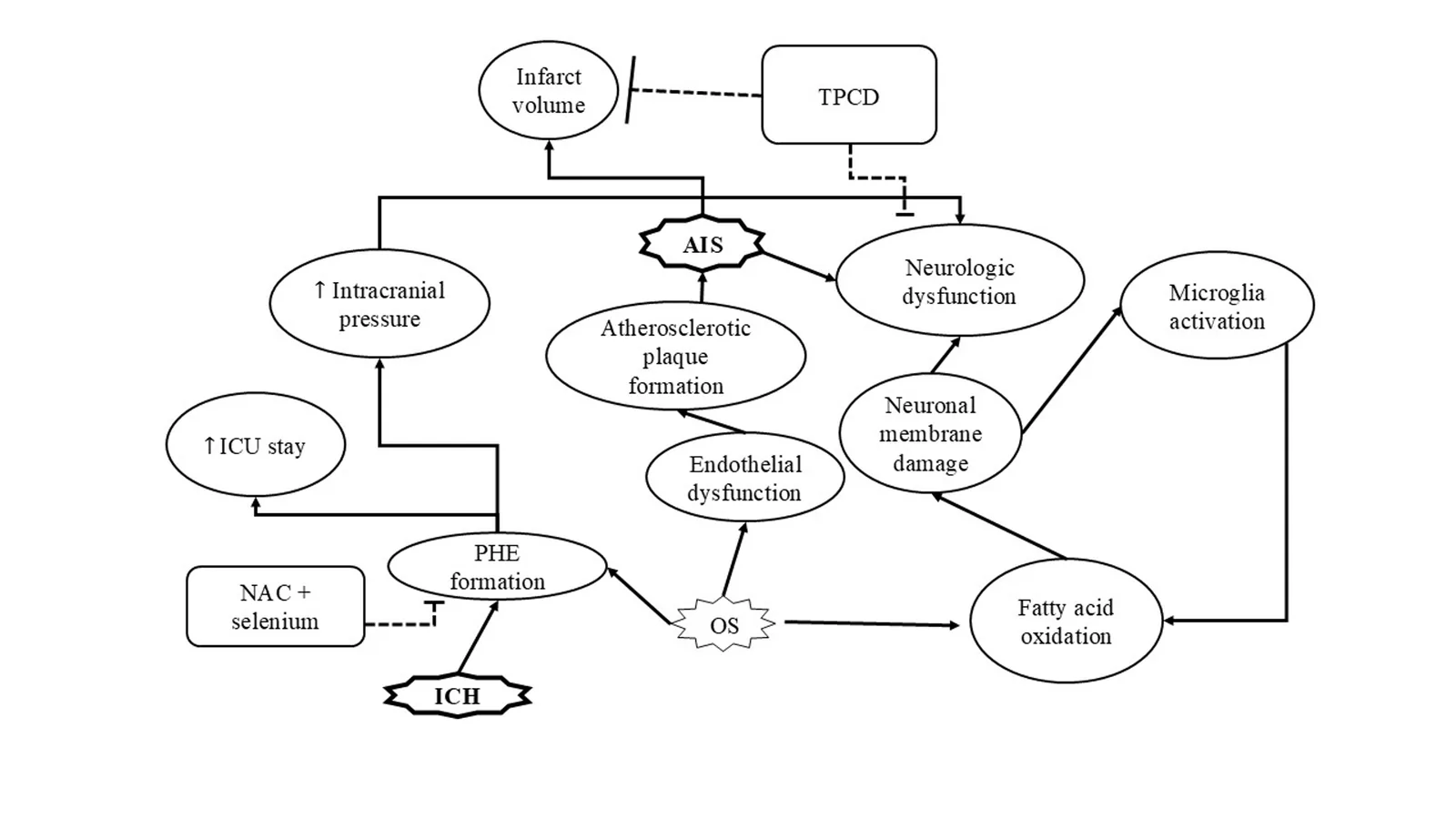
Process diagram of ROS in CeVD (e.g., ICH, AIS) focusing on pathogenesis and therapies Figure created by the authors using PowerPoint (Microsoft, Redmond, Washington). ROS - reactive oxygen species; CeVD - cerebrovascular disease; AIS - acute ischemic stroke; ICH - intracranial hemorrhage; ICU - intensive care unit; NAC - N-acetylcysteine; OS - oxidative stress; PHE - perihematomal edema; TPCD - tempol and a hydrogen-peroxide-eliminating compound of phenylboronic acid pinacol ester onto a cyclic polysaccharide β-cyclodextrin

Cerebrovascular disease (CeVD) refers to a group of neurovascular conditions that manifest due to ischemia of blood vessels supplying the brain [62]. CeVD can be categorized as either ischemic or hemorrhagic, such as events during an intracerebral hemorrhage (ICH). After an ICH, red blood cells are broken down and release hemoglobin. Oxidative damage caused by hemoglobin is a result of Fenton reactions, leading to the production of free radicals. Upon hemoglobin degradation, iron is released, contributing to oxidative stress further. Iron overload following ICH promotes the production of ROS mainly through two pathways: neuronal toxicity through the transferrin receptor system and ferroptosis, an iron-catalyzed lipid peroxidation [63]. In contrast, in the early stage of ischemic stroke, glucose and oxygen shortage enhance ROS production due to mitochondrial damage, leading to the accumulation of superoxide anion, O₂•⁻. Acidification caused by hypoxia further favors the reduction of O₂•⁻ into hydrogen peroxide (H₂O₂) and hydroxyl radicals, which are strongly reactive [64]. CNS cells are particularly vulnerable to the toxic effects of ROS secondary to their naturally elevated oxidative metabolism, lower levels of antioxidant enzymes, and increased concentration of membranous fatty acids [65,66]. In acute ischemic stroke (AIS), OS in the CNS results in similar effects and mechanisms as atherosclerosis and MIRI, as discussed previously (Figure 1, Figure 3). 

AIS is caused by cerebral ischemia and can be broken down into two types: focal ischemia and global ischemia [67]. Focal ischemia occurs when a specific region of the brain is affected, often due to the occlusion of a cerebral artery [68]. In contrast, global ischemia affects the entire brain and is typically involved in cardiac arrest [69].

Therapies for ROS in CeVD

Following an acute ischemic stroke (AIS), microglial cells produce ROS and proinflammatory cytokines. This excessive production of ROS has been linked to brain blood barrier (BBB) disruption, allowing plasma proteins and inflammatory mediators to enter the brain parenchyma. To demonstrate ROS's role in BBB disruption, studies on ischemia-induced-NOX2 knockout mice reported less swelling compared to wild-type controls [70]. Li et al. report that recent clinical trials have shown limited success for most antioxidants in treating chronic inflammatory diseases. This is largely due to issues such as nonspecific distribution, rapid kidney clearance, poor tissue permeability (e.g., across the BBB), and low retention at disease sites such as atherosclerotic plaques [71].

Nonetheless, in a prospective, multicenter, randomized study, the effects of NAC (2000 mg/d) and selenium (1600 µg/d) were assessed in patients with acute intracerebral hemorrhage (ICH). The results demonstrated a significant reduction in perihematomal edema (PHE) volume in the ROS scavenger group compared to the placebo group, and enhanced cognitive recovery following ICH. Additionally, ROS scavenger-treated patients had lower intensive care unit lengths of stay and times, suggesting that early, high-dose ROS scavenger treatment can facilitate improved outcomes in acute ICH [72]

Chen et al. infer that the biomedical effectiveness of nanotherapeutics is largely a consequence of the positive physicochemical and biopharmaceutical properties. These include ultrasmall particle size (usually within the range 10-100 nm), good drug-loading capacity and yield, and enhanced transmembrane permeation allowing for deeper tissue penetration. Nanotherapeutics also protect labile drugs from biodegradation, enable controlled or sustained release through bio-responsive processes, and reduce dosing frequency by maintaining effective drug concentration at targeted sites. They also deliver the drug to specific organs or tissues and improve the overall pharmacokinetic profile of the therapeutic agent [73]. When Tempol and a hydrogen-peroxide-eliminating compound, phenylboronic acid pinacol ester, were incorporated into a β-cyclodextrin-based cyclic polysaccharide nanoparticle (TPCD NP), potent antioxidative, anti-inflammatory, and anti-apoptotic effects were observed after IV administration. In mice with induced acute ischemic stroke (AIS), TPCD NPs accumulated in brain tissue, reduced infarct volume, and improved neurological function. The TPCD NPs served as ROS-sensitive carriers, delivering therapeutic agents to the injury site, and demonstrated good safety in long-term use, highlighting their potential for precision treatment in AIS (Figure 1, Figure 3) [74].

OS plays a central role in the pathophysiology of numerous cardiovascular diseases. Advancements in this field have led to the development of novel redox therapies, including targeted mitochondrial agents and ROS-responsive biomaterials. These therapies are designed to regulate ROS and mitigate oxidative damage to cardiac tissue. While these therapies show promise in preventing and treating cardiovascular diseases, there is a lack of human studies proving their clinical benefits. Nevertheless, advancements in redox therapy may enhance cardiovascular outcomes and the integration of precision medicine in redox-based therapeutic strategies.

ROS in hematological disease

At low levels, ROS can trigger hematopoiesis via stimulation of erythropoietin (EPO) synthesis by the kidneys. However, excess ROS can cause damaging effects to the DNA, proteins, and lipids of hematopoietic stem cells (HSCs). In the reticulocyte stage, excessive ROS can lead to membrane fragmentation and apoptosis, exhibiting characteristics similar to aplastic anemia. Additionally, OS can impair the maturation of reticulocytes. When under conditions of erythropoiesis, ROS act as signaling molecules, but when ROS are in excess, oxidative damage occurs and disrupts the process of autophagy that participates in reticulocyte maturation. The result is ineffective erythropoiesis. Dysfunction in erythropoiesis, particularly under conditions of OS, significantly contributes to the development and exacerbation of hematological disorders [75].

ROS in Anemia

**Figure 4:**
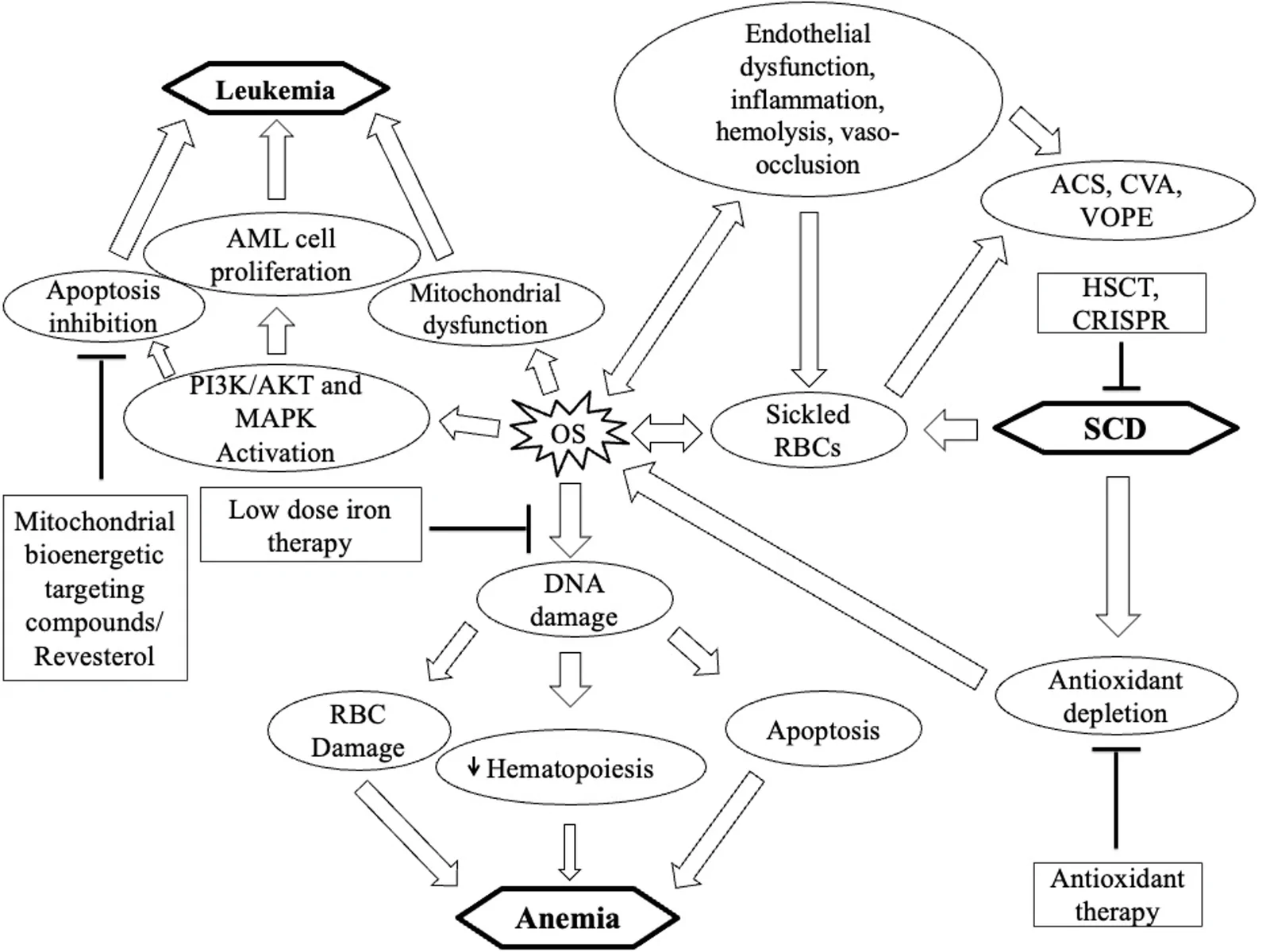
Organizational chart of ROS in hematologic diseases focusing on pathogenesis and therapeutics Figure created by the authors using PowerPoint (Microsoft, Redmond, Washington). ROS - reactive oxygen species; ACS - acute coronary syndrome; AML - acute myelogenous leukemia; CRISPR - clustered regularly interspaced short palindromic repeats; CVA - cerebrovascular accident; HSCT - hematopoietic stem cell transplant; MAPK - mitogen-activated 23 protein kinases; OS - oxygen stress; PI3K/AKT - phosphatidylinositol 3-kinase/protein kinase B; RBC - red blood cell; SCD - sickle cell disease; VOPE - vaso-occlusive pain episode

Specifically, OS plays a role in the pathogenesis of anemia, a condition characterized by a reduced number of red blood cells (RBCs) due to various causes such as blood loss, iron deficiency, and erythropoiesis [76]. While ROS are necessary for erythropoiesis, excessive ROS production can lead to OS and interference with DNA replication and transcription, leading to mutations and genomic instability [7]. In anemias such as Diamond-Blackfan anemia, OS causes DNA damage in erythroid progenitor cells, leading to cellular senescence and activation of the DNA damage response. This results in apoptosis of developing erythroblasts and altered membrane characteristics of mature erythrocytes, including reticulocytes, which are then prematurely destroyed by macrophages [10]. Moreover, RBCs are prone to oxidative damage by virtue of their oxygen burden and hemoglobin's propensity to autoxidize and generate ROS [77]. The ROS would lead to membrane damage of the RBC, reducing its flexibility and increasing rigidity, eventually leading to macrophage-mediated removal of the damaged cells (Figure 4) [78].

Therapies for ROS in Anemia

Redox therapies targeting ROS-mediated damage to RBCs are numerous. One study investigated the effects of two antioxidant cocktails to treat thalassemia/hemoglobin E, including combinations of hydrophobic and hydrophilic antioxidants with an iron chelator. The first cocktail consisted of curcuminoids, NAC, and deferiprone, and the second cocktail consisted of vitamin E, NAC, and deferiprone. Curcuminoids are outfitted with a diketone group, which enables them to directly bind free iron and modulate the expression of proteins involved with iron depletion, such as ferritin, transferrin receptors, and iron regulatory proteins. Similarly, deferiprone binds iron, mitigating iron overload and OS. On the other hand, vitamin E works by scavenging ROS directly and protects cell membranes from oxidative damage. The investigators found that both cocktails significantly improved patients' anemia, reduced OS, and hypercoagulability. However, the vitamin E cocktail had a slightly greater reduction in serum ferritin and non-transferrin-bound iron (NTBI) levels compared to the curcuminoids cocktail [79].

In RASA3-mutated mice, increased levels of ROS caused cell cycle defects and impaired terminal differentiation of reticulocytes during erythropoiesis, which closely resembled anemia but without evidence of increased apoptosis [80]. Many endogenous enzymes responsible for eliminating excess ROS use iron as a cofactor. When levels of iron are low, the body's antioxidant defense system is vulnerable to OS. Iron is also essential for the optimal functioning of the ETC within mitochondria, which becomes another source of ROS when iron levels are inadequate. Iron replacement therapy has been proven to reduce ROS-mediated damage to DNA repair mechanisms in peripheral leukocytes. Iron-sulfur clusters also serve as essential cofactors for numerous enzymes involved in DNA replication, repair, and metabolic pathways linked to OS regulation [81]. Hepcidin, a key regulator of iron absorption, has been reported to have an inverse relationship with erythropoiesis in mouse models [82]. In a separate study on rat models, a significant rise in hepcidin occurred after an iron-induced increase of ROS [83]. Furthermore, DNA lesions accompany the functional loss of hematopoiesis (Figure 4) [84]. In the setting of acute blood loss, there are markedly reduced levels of glutathione (GSH), reflecting an increase in ROS production with lower levels of RBCs and hemoglobin [85]. One study assessed DNA damage, OS, and antioxidant markers in children with iron deficiency and found greater DNA damage in both the iron deficiency and related anemia groups compared. However, low-dose iron therapy led to a significant reduction in DNA damage and an improvement in antioxidant status (Figure 4) [81]. These findings all indicate that OS and its effects contribute to the absence of erythrocytes in anemia patients and may be potential targets for therapeutic interventions [86]. ​​ 

ROS in SCD

SCD, a type of anemia, is a genetic disorder characterized by the production of sickle-shaped RBCs that can block blood vessels and cause tissue damage. At baseline, sickle RBCs generate ROS through the auto-oxidation of sickle hemoglobin and the activation of NADPH oxidase [87]. Patients who receive frequent blood transfusions - such as patients with SCD who suffer from chronic anemia - are prone to having increased levels of ROS from iron-catalyzed reactions (e.g., Fenton reaction), causing endothelial dysfunction and vaso-occlusive crisis (VOCs) [88]. Patients with SCD often experience hemolysis, which releases heme into the plasma. The heme intercalates within endothelial cell membranes and generates ROS in the form of hydroxyl radicals. Elevated ROS can lower cellular levels of NO, a strong vasodilator, inducing vasoconstriction and increasing the risk of VOCs. Additionally, NO is converted to peroxynitrite, a powerful oxidant, exacerbating systemic OS. Several endogenous pro-oxidant enzymes are upregulated in those with SCD, such as NOX and XO. Lipids that are peroxidized by ROS can create reactive aldehydes (e.g., 4-hydroxy-2-nonenal), which can bind proteins that alter cellular signaling and membrane integrity. ROS can also cause post-translational modifications (PTMs) to proteins on the surface of RBCs, impairing their function [89]. This range of effects that ROS have on the pathogenesis of anemia and SCD showcases complexity and potential for therapeutic exploration (Figure 4).

Therapies for ROS in SCD

Oxidative damage, in the setting of SCD, exacerbates hemolysis and promotes vaso-occlusion, creating a vicious cycle of inflammation and further ROS production [90]. SCD-related complications can be triggered by ROS-induced endothelial dysfunction and inflammation [88]. In vaso-occlusive pain episodes, such as cerebrovascular accidents, the pathophysiology involves polymerization of deoxygenated hemoglobin, causing vaso-occlusion in their respective locations [91]. Furthermore, key antioxidant enzymes are significantly diminished in SCD, leading to an imbalance between pro-oxidants and antioxidants (Figure 4) [92]. The use of antioxidants, such as NAC, has shown promise in ameliorating OS and improving clinical outcomes in SCD patients. NAC increases glutathione levels, reduces OS markers, and decreases phosphatidylserine exposure on RBC membranes [93].

Furthermore, iron chelators such as deferoxamine and deferasirox have demonstrated antioxidant properties through scavenging free iron species. Deferoxamine reduces morbidity and mortality by preventing oxidative damage to vital organs before being eliminated by the kidneys via a phenomenon termed the "shuttle hypothesis" [94]. This type of combination therapy could potentially provide therapeutic benefits to patients with toxic levels of iron. In addition to antioxidant therapy, allogeneic hematopoietic stem-cell transplantation (HSCT) has proven beneficial for treating SCD. In this procedure, HSCs are infused into a patient to restore bone marrow function, replacing a defective or cancerous blood-forming system with healthy stem cells. This allows recovery of normal blood cell production and immune function. Unfortunately, this procedure does not come without complications, which could include infection, graft rejection, or graft-versus-host-disease (GVHD) [95]. Moreover, in a study of 1000 SCD patients who received an HSCT, a 93% overall survival rate was reported. Thus, HSCT can result in a high rate of disease-free survival for individuals with SCD (Figure 4) [96].

ROS in leukemia

Leukemia is a condition characterized by the overproduction of abnormal leukocytes [97]. ROS play multiple roles in the pathogenesis of leukemia, from fueling its development to inhibiting its growth. Evidence shows that ROS can induce apoptosis in leukemic cells via both extrinsic and intrinsic pathways. The extrinsic pathway involves ROS stimulating receptors such as Fas and TNFR1, which causes the formation of the death-inducing signaling complex and the activation of caspase-8. By comparison, the intrinsic pathway involves mitochondrial dysfunction with the release of cytochrome c and the activation of caspase-9, which then activates caspase-3. During OS, leukemic cells upregulate their antioxidant defense systems (e.g., SOD, catalase, GPx) to combat the damaging effects of ROS. These endogenous antioxidants act as first-line defenses against ROS and prevent their harmful effects [98]. ROS can also stimulate the NF-pathway, which upregulates anti-apoptotic proteins such as BCL-2 [11,99].

Leukemic progression can be promoted through ROS interactions with MAPK signaling pathways, including extracellular signal-regulated kinase (Erk), c-Jun N-terminal kinase (JNK), and p38. Survival pathways that cross-talk with MAPKs, including PI3K/Akt, also contribute to leukemic progression and resistance to apoptosis when activated by ROS [97]. Elevated intracellular ROS levels activate ERK-dependent pathways, leading to NF-κB phosphorylation and promoting the proliferation of acute myeloid leukemia (AML) cells [99]. In normal physiology, JNK and p38 have been noted for their stress-activated induction of apoptosis [100]. In the setting of malignancy, JNK genes also play a central role in metastasis through the regulation of inflammation, angiogenesis, cell migration, and intravasation. Within the tumor microenvironment, they stimulate epithelial-to-mesenchymal transition by facilitating the release of growth factors [101]. IL-33 was observed to promote survival and cell cycle progression by activating p38 [102]. By inactivating protein phosphatases (PTPs), ROS further enhance leukemic proliferation by preventing MAPK dephosphorylation and sustaining their active state. ROS-induced damage to oncogenic genes, such as RAS, can lead to constitutive MAPK activation, driving leukemia progression [103]. ROS can also affect cellular mitochondria in leukemia, as mitochondria play a key role in the apoptotic process of cells. Mitochondria contain BCL-2 proteins, which are regulators of apoptosis by preventing mitochondrial outer membrane permeabilization, thereby blocking the release of cytochrome c and activation of the caspase cascade (Figure 4) [104].

Therapies for ROS in Leukemia

Redox therapy in the treatment of leukemia involves both antioxidants and pro-oxidants. Resveratrol, a powerful antioxidant and anti-cancer medication, demonstrates anti-cancer effects by inducing cell cycle arrest, apoptosis (via Bax, Bak, caspase-3/9), and OS, while inhibiting angiogenesis and inflammation. These pathophysiological mechanisms collectively suppress tumor growth and enhance cancer cell death, making resveratrol a promising therapeutic agent in the treatment of myelogenous and lymphoblastic leukemia (Figure 4) [105]. Redox therapies under investigation to treat leukemia through pro-oxidative effects include ellagic acid, acacetin, arsenic trioxide (ATO), and decitabine (DAC). Ellagic acid has been found to induce apoptosis in chronic lymphocytic leukemia (CLL) cells by acting as a pro-oxidant in mitochondria, increasing ROS formation and triggering mitochondrial swelling, cytochrome c release, and caspase 3 activation [106,107]. Similarly, acacetin acts as a pro-oxidant. It has been found to induce apoptosis in CLL cells by increasing ROS formation in mitochondria, leading to diminished mitochondrial membrane potential and release of cytochrome c [108]. ATO, a US Food and Drug Administration (FDA)-approved medication, is used to treat acute promyelocytic leukemia (APL), resulting in differentiation of leukemic promyelocytes [109]. Furthermore, immunohistochemistry analysis on human fibroblasts demonstrated an increase in OS in ATO-treated groups [110]. DAC, an FDA-approved medication for the treatment of MDS and AML, appears to increase ROS accumulation within leukemia cells, triggering cell cycle blockage and apoptosis [109]. Thus, these compounds selectively induce apoptosis in leukemia by increasing ROS production, disrupting mitochondrial functions, and activating apoptotic pathways (Figure 4).

An experimental phytochemical found in ginger, 6-shogaol, demonstrates antioxidant and anti-inflammatory activity while also having hematopoietic, hepatoprotective, and anti-cancer effects in MDS patients. Studies have found it to reduce the production of proinflammatory cytokines and inflammation by altering several signaling pathways, including the NF-, activator protein, MAPK cascades, and peroxisome proliferator-activated receptor gamma. The antioxidant nature of 6-shogaol stems from being outfitted with alpha, beta, and unsaturated ketone moieties that scavenge free radicals, causing less lipid peroxidation and XO activity. 6-shogaol also stimulates the nuclear factor-erythroid 2-related factor 2 signaling pathway, which upregulates the expression of phase II antioxidants such as GSH [111]. Therefore, ROS play an essential role in initiating signaling cascades that are responsible for leukemic cell survival and proliferation, confirming that ROS have a double-sided influence on the development of leukemia.

ROS in hemophilia

Hemophilia is an X-linked genetic bleeding disorder caused by deficiencies in clotting factors, including both hemophilia A from a lack of factor VIII and hemophilia B from a lack of factor IX [112]. Recurrent bleeding occurs primarily in the joints and muscles, leading to pain, swelling, and potential compartment syndrome, which may require surgical intervention. OS is reported to exacerbate the complications associated with hemophilia in multiple ways. For example, patients with hemophilia are susceptible to prolonged and excessive bleeding from minor trauma or spontaneously [113]. 

**Figure 5:**
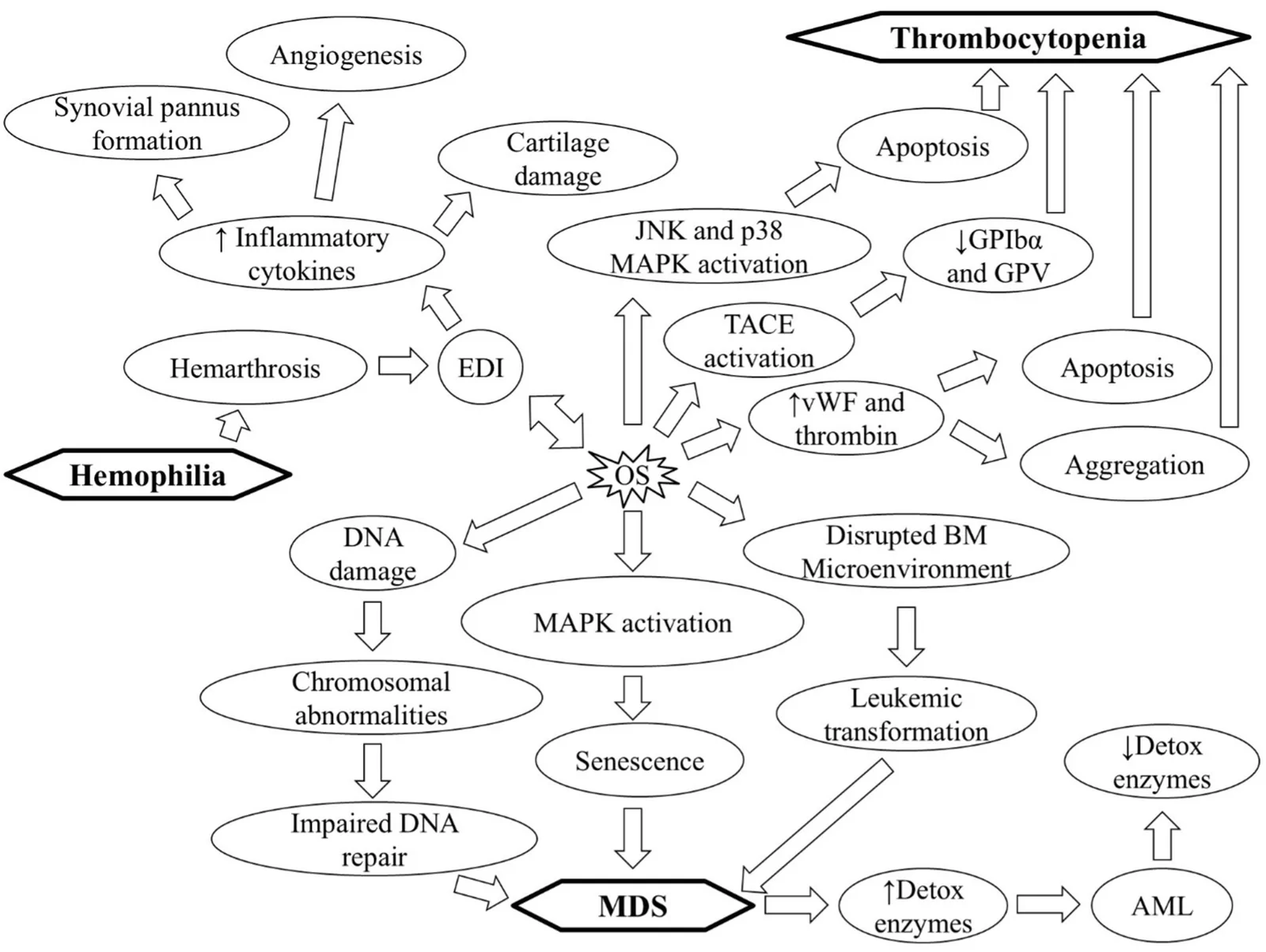
Organizational chart of ROS in hematologic diseases focusing on pathogenesis and therapeutics Figure created by the authors using PowerPoint (Microsoft, Redmond, Washington). ROS - reactive oxygen species; AML - acute myeloid leukemia; BM - bone marrow; DNA - deoxyribonucleic acid; EDI - erythrocyte-derived iron; JNK - c-Jun N-terminal kinase; MAPK - mitogen-activated protein kinases; MDS - myelodysplastic syndrome; OS - oxygen stress; TACE - tumor necrosis factor-α-converting enzyme; vWF - von-Willebrand factor

Iron within the joint spaces undergoes the Fenton reaction to form highly reactive hydroxyl radicals that have damaging effects on lipids, proteins, and DNA of cells within joint spaces. This can result in chondrocyte death, joint inflammation, and cartilage breakdown, all of which are key contributors to the development of chronic hemophilic synovitis and arthropathy [114]. In a study on induced iron production in human monocyte cells, the addition of heme was found to enhance inflammatory responses by increasing the activation of NF-κB and the expression of proinflammatory cytokines [115]. Additionally, erythrocyte-derived iron generates harmful hydroxyl radicals when interacting with H2O2, which cause cellular damage and permanent cartilage destruction. This leads to angiogenesis, increased inflammatory cells, and cartilage damage in the joint space, creating a cycle of bleeding and inflammation (Figure 5) [113].

Another literature review showed that erythrocyte-derived iron and interleukin-1 beta (IL-1β)-induced H2O2 contribute to the direct destruction of the cartilage matrix. This is through inducing the formation of ROS and reacting with hemoglobin-derived iron to form hydroxyl radicals [112].

Therapies for ROS in Hemophilia

Researchers tested both formulations on human synovial fibroblasts and found that they reduced TNF- and IL-6 levels within articular macrophages and only IL-6 in synovial cells [116]. Curcumin is equipped with chemically active keto/enol groups that have powerful antioxidant effects. Curcumin activates nuclear factor erythroid 2-related factor 2 (Nrf2), which binds to antioxidant response elements and enhances the transcription of antioxidant enzymes such as SOD, GPx, and CAT. The combined effects of curcumin could potentially alleviate joint damage in patients with hemophilia and improve their quality of life [117]. Thus, hemophilia is markedly impacted by OS through many distinct pathways.

ROS in thrombocytopenia

Thrombocytopenia is a condition where platelet count drops below 150 × 10³ per μL, usually caused by factors such as decreased production, increased destruction, splenic sequestration, or platelet dilution/clumping [118]. In the pathogenesis of thrombocytopenia, ROS are involved in the regulation of platelet function and activation [119]. Increased ROS levels are responsible for causing overactivation of platelets due to increased platelet activator levels. The platelet activators von Willebrand factor (vWF) and thrombin are the factors responsible for causing a procoagulant phenotype and apoptosis, which correlate with thrombocytopenia [120]. In the pathogenesis of thrombocytopenia, ROS are involved in the regulation of platelet function and activation [119]. Increased ROS levels are responsible for causing overactivation of platelets due to increased platelet activator levels. The platelet activators von Willebrand factor (vWF) and thrombin are the factors responsible for causing a procoagulant phenotype and apoptosis, which correlate with thrombocytopenia [120]. Brill et al. demonstrated that oxidative damage activates tumor necrosis factor-α-converting enzyme (TACE) in mice and in vitro experiments, leading to the shedding of key platelet receptors, GPIbα and GPV. This process, dependent on p38 MAPK signaling, reduces platelet function and prevents them from incorporating into a growing thrombus [9]. Furthermore, studies correlating OS to thrombocytopenia have shown that mitochondrial ROS-mediated activation of JNK and p38 MAPK induced human platelet apoptosis ex vivo [121]. Thus, the involvement of ROS in thrombocytopenia pathogenesis highlights their significant role in platelet dysfunction, activation, and depletion, and thus in the low platelet count and impaired thrombus formation characteristic of this disorder (Figure 5).

In immune thrombocytopenia (ITP), a form of thrombocytopenia, the immune system produces antibodies against platelets, impairing platelet function and reducing their amount [122]. Using gene expression analysis on the peripheral blood of patients with ITP, researchers reported that the GSH / GSH disulfide ratio was significantly higher in healthy controls than in patients with ITP [123]. When there is insufficient antioxidant scavenging capacity, ROS could damage platelet membranes, resulting in the loss of platelet membrane elasticity, increased membrane fragility, and shortened cell life [124]. ROS also modify protein antigens on the membranes of platelets, making them more immunogenic. The scarcity of regulatory T cells increases the autoreactivity of T and B cells, worsening the autoimmune reaction in ITP. The highly reactive aldehyde, MDA, can be generated through lipid peroxidation catalyzed by ROS, which further damages cells and contributes to the pathogenesis of ITP [124].

Therapies for ROS in Thrombocytopenia

Therapies targeting ROS are becoming an increasingly important approach in treating thrombocytopenia caused by OS, reducing platelet dysfunction and improving the prognosis for patients. Ya et al. reported that protocatechuic acid (PCA) dose-dependently inhibits H2O2-induced human platelet apoptosis by preventing mitochondrial membrane potential dissipation and caspase activation. Further investigation showed that PCA can modulate mitochondrial and cytosolic distributions of Bax, Bcl-xL, and cytochrome c. PCA also suppresses ROS generation and intracellular Ca2+ concentration in platelets, possibly through inhibition of the ROS-dependent PI3K/Akt/GSK3β pathway [125]. These events were observed in vitro, and clinical studies are yet to be reported. In addition, curcumin, labeled by the FDA as generally recognized as safe, is used as an antioxidant and immune activity-modulating agent and has been evaluated for its effects on platelet activity [126]. It was found to protect against OS-induced platelet depletion by inhibiting MAPK activation and restoring apoptotic markers in human platelets ex vivo [119,127].

Studies using resveratrol inhibited platelet function and reduced thrombus formation in patients with type 2 diabetes. Key enzymes involved with glucose metabolism, including hexokinase, glucose-6-phosphate dehydrogenase, aconitase, and isocitrate dehydrogenase, can all be blocked by resveratrol, resulting in reduced platelet activation. Thromboxane, a potent promoter of platelet aggregation, is also reduced by resveratrol. This reduction appeared more prominent in diabetic platelets, suggesting that a hyperglycemic state yields a greater therapeutic effect. Therefore, resveratrol may help reduce platelet function and thrombus formation in type 2 diabetes, making it a potential complementary therapy for preventing vascular complications [128].

ROS in MDS

MDS is a group of hematological disorders characterized by abnormal blood cell production, often leading to anemia, neutropenia, thrombocytopenia, and a higher risk of developing acute myeloid leukemia [129]. The role of ROS in the pathology of MDS is multifaceted. At low physiological concentrations, ROS act as signaling molecules by reversibly oxidizing specific cysteine residues on target proteins, leading to PTMs that regulate protein activity [130]. This redox signaling is involved in numerous cellular processes, including metabolic regulation, stress responses, and adaptation to environmental changes [131]. However, high ROS levels were linked to high levels of serum ferritin and low levels of hemoglobin in MDS, implying that iron buildup or severe anemia may worsen OS [132].

A second pathway of ROS-induced MDS involves the ability of high levels of ROS to inflict oxidative DNA damage on HSCs. The effect of ROS on epigenetic regulators, including DNA methyltransferases and histone deacetylases, can lead to aberrant gene expression and altered regulation of genes responsible for cell cycle control, apoptosis, and differentiation, contributing to the progression of MDS. DNA damage, in turn, gives rise to genomic instability. This process, in part, involves the loss of the protection provided by the FOXO3 transcription factor. This loss further results in acceleration of DNA damage, along with reduced ability for DNA repair, leading to hematological malignancies (Figure 5) [8].

ROS affect the bone marrow microenvironment, influencing HSC self-renewal, migration, and differentiation. Chronic high ROS levels in the bone marrow disrupt the balance between quiescence and activation of HSCs, promoting leukemic transformation (Figure 5) [133]. A study conducted by Picou et al. reported that the expression of ROS-generating enzymes was elevated in the bone marrow of MDS patients. Moreover, the progression from MDS to AML could involve an increase in the expression of detoxifying enzymes; however, upon the progression to acute AML, there is a significant decrease in the levels of these detoxifying enzymes (Figure 5) [134]. The activation of inflammasomes, such as NLR family pyrin domain containing 3, and the production of proinflammatory cytokines were associated with elevated levels of ROS, leading to increased cell death and ineffective hematopoiesis. ROS also influence the growth, self-renewal, and differentiation of HSCs, which are essential aspects of hematopoiesis. Signaling pathways such as NF-, MAPK, and PI3K/Akt, which are key regulators of cell survival, proliferation, and apoptosis, can be altered by ROS, disrupting their function and promoting the onset of MDS (Figure 5) [135].

Therapies for ROS in MDS

Antioxidant therapy, with agents such as amifostine or 6-shagol, has demonstrated promising outcomes in MDS. has been investigated for its therapeutic properties in MDS. For example, when amifostine is absorbed into tissues, it gets dephosphorylated into its active form, WR-1065, which is a potent scavenger for ROS. It also exhibits antimutagenic effects by scavenging ROS and protecting erythroid progenitor cell DNA from oxidative damage. Thus, healthy tissues can be spared from the toxic effects of chemo- or radiotherapy without compromising their therapeutic efficacy. This is especially beneficial for minimizing oxidative damage to healthy HSCs. Amifostine also reduced telomerase activity in AML cells, which is associated with survival and proliferation of malignant cells [136]. The antioxidant phytochemical, 6-shogaol, may reduce the onset of MDS and prevent leukemic transformation through uptake by leukemic cells. The reduction of OS by 6-shogaol may reverse impaired hematopoiesis within the bone marrow of patients with MDS. Thus, targeting ROS and OS pathways with antioxidants may have therapeutic potential in MDS with promising results in preclinical studies [135].

Advancements in nanotechnology and genetic engineering

Nanotechnology and CAD

Nanotechnology is a rapidly advancing field involving the measurement, manipulation, and assembly of matter on a nanometer scale [137]. It is being explored for its utility in the treatment of CAD, the most common form of heart disease and leading cause of death in the United States [138]. The traditional approach to detecting CAD involves measuring cardiac troponins, C-reactive protein, creatine kinase MB, and microRNAs in the bloodstream, which requires costly resources and time. However, nanotechnology appears to be a more efficient and precise method to analyze these biomarkers. Nanotechnology employs various types of biosensing methods to detect biomarkers of CAD, including electrochemistry, electrochemiluminescence, fluorescence, colorimetry, and surface plasmon resonance technology. Nanomaterials' high binding affinities and optoelectronic properties make nanotechnology coupled with biosensing a promising approach for diagnosing CADs [12]. In a study using upconversion nanoparticles (NPs) combined with a lateral flow assay for fluorescence ratiometric detection of myoglobin in clinical blood samples, the optical biosensing apparatus achieved a detection limit as low as 0.21 ng/mL and a detection time of just 10 minutes. This detection limit is reportedly about 50 times lower than comparison studies using different techniques. Furthermore, the results remained unaffected by environmental interferences such as high bilirubin, elevated lipid levels, hemolysis, and various other biomarkers [139].

Current treatments for coronary heart disease (CHD) consist of using drugs to regulate the effects of CHD, such as blood pressure, blood glucose, and inflammation. However, drugs like statins have potential side effects, which may lead to further health implications, such as liver damage. Yet, nanotechnology, using NPs, offers new medical applications in CHD. Moreover, NPs encapsulate multiple small molecules for in vivo drug transport, improving the efficacy of drug delivery. Paul et al. designed an injectable graphene oxide nanosheet-collagen-methacrylamide hydrogel with VEGF-165 plasmid DNA for cardiac repair. The hydrogel was not toxic during gene release in rats, promoted vascular growth, suppressed scarring, and recovered heart function within days. However, human trials are required due to potential risks like fibrosis, premature degradation, and immune reactions [140]. Thus, NPs have several advantages over traditional drugs for early CHD detection, including more advanced biosensing and increased drug delivery efficiency.

CRISPR and Hematological Disorders

Clustered regularly interspaced palindromic repeats (CRISPR-Cas9) is a novel gene-editing technology that has advanced biomedical research, making it possible to correct errors in genomes and turn genes on and off. This system involves two components: a guide RNA (gRNA) and a CRISPR-associated protein 9 (Cas9) endonuclease. This 20-nucleotide gRNA binds to the target DNA sequence and directs the Cas9 protein to create double-stranded breaks at the target site. DNA repair following a cut can be done in two ways: non-homologous end joining (NHEJ) or homology-directed repair. NHEJ is likely to generate random loss or addition of DNA; meanwhile, homology-directed repair uses a homologous DNA template to facilitate precise genome editing [141]. These versatile repair mechanisms enable CRISPR-Cas9 to be used for a wide range of applications, from basic research to therapeutic gene editing. For example, genetic engineering has become a robust approach for treating SCD. The development of SCD is linked to the replacement of glutamic acid with valine in the HBB gene [142]. This mutation results in the production of an abnormal form of hemoglobin known as hemoglobin S (HbS), which polymerizes in hypoxic conditions and OS [143]. This polymerization can cause VOCs, ischemia, and episodes of extreme pain [144].

CRISPR-Cas9 has applications in β-hemoglobinopathies, including SCD and β -thalassemia. The current treatment of SCD uses antineoplastics and long-term blood transfusions [145]. However, allogeneic HSCT, using CRISPR, presents a promising curative alternative to existing treatments, with reported cure potential in >95% in pediatric populations and >90% in adult populations (Figure 4). With that said, hematopoietic stem cell transplant (HSCT) is a high-risk procedure with complications such as graft failure and infection [146]. A mutation in the HBB gene leads to SCD, so ex vivo gene therapy involving the insertion of a modified HBB gene into HSCs could provide long-term treatment for SCD [147].

In one study by Demirci et al., ex vivo fetal hemoglobin (HbF) induction demonstrated promising results. HbF prohibits sickle hemoglobin polymerization, as it is observed that naturally occurring levels of HbF ameliorate SCD symptoms. Thus, reactivating silenced HbF in red blood cells or genetically editing HbF repressors could have therapeutic potential. However, the immune response against in vivo gene editing may hinder the efficacy of this approach [148]. It is important to note that further research is required to better understand its impact in a clinical setting. Traditional treatment options for these complications include hydroxyurea, blood transfusions, opioids, and prophylactic antibiotics. Of the treatment options available, genetically engineered ex vivo allogeneic HSCT appears to be a promising option for achieving remission from SCD. Thus, genetic engineering techniques employed to potentially enhance the therapeutic efficacy of HSCs include gene-addition therapy, gene-editing therapy, and base editing.

Gene-addition therapy has shown significant clinical benefits. Such a therapeutic approach requires extracting HSCs from the patient, genetically modifying them using a BB305 lentiviral vector to add a therapeutic HBB gene, and then reinfusing them back into the patient. In phase 1/2 clinical studies, gene therapy with modified CD34+ cells safely reduced or eliminated the need for long-term transfusions in patients with severe β-thalassemia [149]. Furthermore, in SCD patients, phase 1/2 studies have shown marked improvement in hemoglobin production and even cessation of SCD-related events. However, these findings require confirmation in larger sample sizes [150]. Another gene-editing procedure involves collecting a patient's HSCs and using CRISPR-Cas9 to target the BCL11A gene, resulting in increased HbF production and a decreased frequency of VOCs in preclinical animal models.

CRISPR-mediated gene editing could repair mutations in the Fancf gene and restore the FA pathway in mouse embryonic stem cells [151]. Additionally, Román-Rodríguez et al. eliminated an insertion mutation responsible for a premature stop codon, effectively restoring FANCA gene function without disrupting other regions of the gene. They also demonstrated that NHEJ-mediated gene editing could successfully restore gene function in CD34+ cells derived from FA patients [152]. Hence, using CRISPR as a potential therapeutic strategy for patients with SCD and Fanconi anemia holds great potential.

Compared to CRISPR, base editing is a recently developed, more precise genome editing technique that utilizes the CRISPR system. Instead of causing double-strand breaks (DSBs), base editors employ a catalytically inactive or nickase Cas protein with a deaminase enzyme fused onto it [153]. Base editing does not produce DSBs and therefore tends to reduce the risk of large indels and chromosomal rearrangements. However, base editors are not without risk either, including off-target DNA and RNA deamination, bystander editing within the editing window, and, in certain cases, low-frequency DSBs or genotoxic byproducts such as deletions and translocations, though the latter are counted at lower frequencies than with nuclease-based CRISPR editing [154].

The application of gene-addition therapy, gene-editing therapy, and base editing has proven to be a promising avenue of treatment for SCD. Leveraging the use of genetic engineering to enhance the efficacy of redox therapy may create groundbreaking treatment options for patients with SCD. However, more investigation is needed to understand the most optimal combination of approaches.

Future prospects of redox therapy

Redox therapy is a promising approach for the treatment of cardiovascular and hematological diseases. Investigation into the connection between OS and ROS-sensitive cellular signaling molecules also holds promise for preventing the onset of complications from diseases. Understanding the complex interactions of ROS dysregulation can inform the development of new therapeutics that alter ROS levels and treat diseases. Redox therapies have proven to alleviate OS in patients and animal models with cardiovascular disease by decreasing ROS. Additionally, they have also proven to be effective in hematological diseases through similar mechanisms and through OS inducing agents. Nanotechnology has also shown benefit in AIS models by reducing infarct volume and improving neurological function. Despite complications, such as GVHD with genetic engineering for the treatment of SCD in combination with redox therapy could be an effective approach to long-term disease control. Advancements in redox therapy, nanotechnology, and genetic engineering have yielded novel therapeutic options for patients with cardiovascular and hematologic diseases through targeted therapy. However, further investigation of redox mechanisms and other therapies, especially in human trials, is encouraged.

## Conclusions

Collectively, these findings underscore the significance of OS in a range of cardiovascular and hematologic diseases. Excessive ROS production plays a critical role in the onset and progression of these diseases, making it a high-value therapeutic target. Because of the recognition of ROS as an inducer of disease etiology, therapies for preventing and managing OS have been actively developed. These therapies have a main focus on antioxidants, mitochondrial agents, and gene-editing strategies to mitigate disease progression and improve patient outcomes. Additionally, nanotechnology and genetic engineering could augment redox therapy through earlier detection of disease and improved therapeutic efficacy, yet most of the therapies being explored in this field have yet to prove their clinical efficacy. Other aspects-indications, invasiveness, side effects, and long-term outcomes-are also to be carefully weighed. These together highlight the necessity for further research and the promising potential of future treatments for curbing oxidative stress-mediated diseases.
